# Factors Associated with Post-Intensive Care Syndrome in Patients Attending a Hospital in Northern Colombia: A Quantitative and Correlational Study

**DOI:** 10.3390/nursrep15090311

**Published:** 2025-08-25

**Authors:** Jorge Luis Herrera Herrera, Yolima Judith Llorente Pérez, Edinson Oyola López, Gustavo Edgardo Jiménez Hernández

**Affiliations:** 1 Nursing Program, Universidad de Córdoba, Córdoba 230000, Colombia; yllorente@correo.unicordoba.edu.co (Y.J.L.P.); gustavojimenezh@correo.unicordoba.edu.co (G.E.J.H.); 2 Nursing Program, Universidad del Sinú, Córdoba 230000, Colombia; edinsonoyola@unisinu.edu.co

**Keywords:** signs and symptoms, critical care, nurse’s role, survivors, intensive care units, syndrome

## Abstract

**Background/Objectives:** We identified the factors related to post-intensive care syndrome in a sample of patients from northern Colombia. **Methods:** This study employed a quantitative, observational, descriptive, and correlational approach. A sample of 277 adults was obtained through non-probabilistic convenience sampling, and a characterization form comprising sociodemographic and clinical variables was applied. The Healthy Aging Brain Care Monitor (HABC-M) instrument was also used, which is a clinical tool with a high capacity to detect post-intensive care syndrome (PICS) in surviving intensive care unit (ICU) patients. **Results:** The final sample consisted of 277 adults, 67.5% male, with university degrees, cohabiting in a marital union, working, from urban areas, and of the Catholic religion. Seventy percent of the sample presented both cardiovascular and neurological alterations and was admitted to the ICU, and 66% had a personal history of arterial hypertension (AHT) and type 2 diabetes mellitus (DM2). Patients had a mean ICU stay of 10.7 days, with a standard deviation of 4 days, and displayed a moderate risk of morbidity and mortality according to Acute Physiology and Chronic Health Evaluation II (APACHE II). A total of 38.6% of the sample received mechanical ventilation, with a mean duration of 8.3 days, and 7.5% underwent tracheostomy. As for sedation, 38.6% were administered fentanyl. In total, 83.4% of the sample presented the syndromes under study, with a predominance of the severe category. The global score of the scale was taken as the dependent variable, and statistical significance (*p* < 0.05) was found with sociodemographic variables, including origin and religion, and with clinical variables such as receiving pharmacological treatment. **Conclusions:** The sample presented PICS globally and showed how it affects the different dimensions, showing associations with the sociodemographic and clinical variables of interest.

## 1. Introduction

The definition of post-intensive care syndrome (PICS) dates back to 2012, when experts from the Society of Critical Care Medicine (SCCM) proposed this term to group a set of cognitive, psychological, and physical symptoms that occur after a critical illness [1]. Furthermore, the SCCM recommended that this syndrome should not be approached as a medical diagnosis but as a concept aimed at improving education and awareness of post-discharge deterioration from an intensive care unit (ICU) [1].

Once PICS was recognized as a consequence of the care process in critically ill patients, research such as the ARDSNet Long-Term Outcomes Study (ALTOS) documented that, during the first year after discharge from the ICU, more than two-thirds of survivors reported clinically significant symptoms of fatigue, in addition to manifestations of anxiety and depression [2]. Similarly, Geense et al. not only confirmed the findings reported by the ALTOS regarding PICS symptoms but also identified that health status prior to admission to intensive care was significantly associated with health problems after discharge [3].

Along the same lines, authors such as Hiser et al. highlight other aspects of this syndrome, such as chronic pain, gait disturbances, and difficulty returning to work [4]. The need to understand PICS and improve the care provided to those who suffer from it has generated a growing literature that offers a broad conceptualization of this phenomenon, without overlooking the fact that its influence, due to its various personal and clinical factors, makes its approach complex [5].

The literature indicates that the decrease in mortality rates in intensive care units (ICU) seems to be related to the accelerated incidence of post-intensive care syndrome (PICS) [6]. This is justified by the fact that the greater the number of people who survive in an ICU, the greater the sequelae of critical illness [7,8]. Undoubtedly, this syndrome represents a challenge in medical care. However, the current research indicates that several of the factors influencing the pathophysiology of PICS are modifiable and require continuous monitoring by the healthcare team [9,10].

In terms of epidemiological behavior, countries with higher per capita incomes, such as Spain [11], South Korea [12], and France [13], have reported incidences of PICS between 40 and 50%, with predominantly psychological involvement. When contrasting these data with those reported in countries with lower levels of economic income, the prevalence of PICS does not seem to be determined by the degree of economic development, as similar incidence rates have been documented in countries such as Chile [14], Brazil, and Colombia [15].

Another relevant aspect of PICS is its risk factors. In this regard, Colbenson et al. [16] pointed out that the use of sedoanalgesia, metabolic alterations such as hyper- and hypoglycemia, and hypoxia are associated with the onset of PICS. Hatakeyama and colleagues [17] identified a relationship between prolonged mechanical ventilation and the diagnosis of delirium during ICU stays, with a higher incidence of this syndrome. Likewise, some sociodemographic variables, such as sex, age, and the presence of comorbidities, have also been shown to be related to the development of PICS, being more frequent in the elderly and those with pre-existing diseases [18].

A review of the current knowledge, which, as mentioned above, has increased in recent years, provides a clear picture of the determinants of post-intensive care syndrome (PICS) and the impact that this set of signs and symptoms can have on the health of individuals during their hospital stay and after discharge from the ICU. However, given that it is conceptualized as a multifactorial and multidimensional entity with a high incidence, further research is needed to strengthen our understanding of this phenomenon in order to propose interventions to prevent it [19].

Colombia, for its part, is no stranger to the reality described above. A study carried out in Bogotá classified the severity of acute postoperative syndrome (PAS) into three levels: mild, moderate and severe. The main factors associated with the moderate and severe categories were male sex, APACHE II score, age, length of stay in the ICU, and the use of sedoanalgesia and neuromuscular relaxants [20]. However, it is necessary to continue proposing research in the Colombian population to provide updated information on the incidence of this syndrome and the predisposing factors in this specific context. Similarly, it remains essential that this phenomenon be analyzed more broadly in Latin America, not only by analyzing its epidemiological behavior and risk factors, but also by verifying the effectiveness of interventions for its prevention and designing or adapting evaluation instruments.

According to the above, there is evidence of a problem of significant social relevance, which is demonstrated by situations in which the sequelae of PICS have prevented employed, active people from returning to their work activities, even one year after hospital discharge. This situation not only decreases the quality of life of the patient, their family, and caregivers but also results in a loss of labor force for society [21]. Likewise, family members may experience symptoms of stress, anxiety, and depression [6], in addition to facing financial tensions and the need to reorganize their priorities, which frequently results in the abandonment of employment or reduced working hours [21].

As far as health systems are concerned, this problem is associated with a significant increase in the direct costs related to healthcare, such as prolonged hospital stays, home care requirements, admission to chronic care units, use of assistive technology devices, and rehabilitation services. Likewise, indirect or non-medical costs increase, including expenses for food, transportation, lodging, and loss of work productivity [22].

In nursing practice, preventing and identifying PICS can be especially challenging given that the wide variety of clinical manifestations may be masked by other complications arising from the ICU care process. This situation can lead to care planning that does not adequately address the needs generated by the human response to the symptoms of PICS. Therefore, it is essential for nursing to assume an active role in the approach to this phenomenon to understand its behavior and find the necessary inputs to propose new research approaches or interventions that effectively respond to the problems described. In this context, the present research was proposed to identify the factors related to post-intensive care syndrome in a sample of patients from northern Colombia.

## 2. Materials and Methods

### 2.1. Study Design

A quantitative, observational, descriptive, and correlational study was conducted following the STROBE guidelines to ensure transparency and completeness in the development of epidemiological studies [23]. This design was used to determine the relationship between variables without the direct intervention of the researcher, allowing estimates to be made between sociodemographic and clinical characteristics and the presence of PICS. Thus, it was feasible to obtain information on the magnitude and direction of the correlations identified, making necessary contributions to improve our understanding of this phenomenon and providing evidence for further research in clinical practice. The use of non-probabilistic convenience sampling was determined by the logistical and ethical limitations of accessing ICU survivors within a defined post-discharge period. While this approach allowed efficient recruitment and operational feasibility, it introduces limitations in the external validity of findings. Specifically, convenience sampling may lead to selection bias, since participants may not be representative of the broader population of ICU survivors. Factors such as greater availability, higher functional capacity, or specific motivations to participate may skew the sample characteristics.

Furthermore, since the variables in this type of design are not manipulated, it is not possible to infer causality. Therefore, the findings must be interpreted as associative, and any observed relationships should be explored further through longitudinal or experimental designs.

### 2.2. Scope and Period

The study was conducted in the hospitalization service of a high-complexity healthcare institution in Córdoba, Colombia. Data collection occurred from August to November 2024. Instruments were applied either during hospitalization or at the patient’s home, depending on discharge status. For home visits, a standardized operating protocol was followed, including prior telephone scheduling, informed consent procedures, infection control measures, and verification of data completeness and reliability, as endorsed by the institutional ethics committee.

### 2.3. Population Sample and Sampling

The target population of this study consisted of adult ICU survivors. We included participants over 18 years of age who had been hospitalized for seven days or more and who provided informed consent. Patients with documented cognitive impairment prior to hospitalization, with a diagnosis of dementia, with an inability to respond to the questionnaire, or who were readmitted to the ICU within 48 h of discharge were excluded. The sample size was determined a priori using G*Power v3.1.9.7. A *t*-test for Pearson correlation coefficients was used with the following assumptions: α = 0.05, β = 0.20 (power = 0.80), and r = 0.20 (small effect size). The estimated minimum sample was 193, adjusted to 251 to account for potential 30% loss. Ultimately, 277 participants were included, yielding a post hoc power > 96% for r = 0.20 and >99% for r ≥ 0.25.

A total of 370 patients were selected, of which 93 were excluded: 62 for not meeting the inclusion criteria, 11 for not providing informed consent, and 20 for other reasons that prevented their participation. The final sample consisted of 277 patients.

### 2.4. Variables and Instruments

#### 2.4.1. Characterization Sheet

Ad hoc design instrument, specifically designed for the collection of sociodemographic variables (age, gender, marital status, educational level, and employment status) and clinical variables (admission diagnosis, comorbidities, APACHE II scale score, days of mechanical ventilation, length of stay in the ICU, and use of sedation)

#### 2.4.2. Healthy Aging Brain Care Monitor (HABC-M)

We used a self-completed instrument, validated in Spanish, with adequate internal consistency (α = 0.83) and construct validity for the assessment of PICS [24]. The scale is composed of 27 items distributed in three domains: cognitive, functional, and psychological. Each item is scored on a Likert-type scale from 0 to 3, where higher scores reflect greater impairment. The cognitive subscale includes six questions related to memory, orientation, and judgment. Its interpretation is based on the following ranges: ≤4 (normal), 5–8 (mild), 9–11 (moderate), and 12–18 (severe). The functional subscale consists of 11 questions oriented to assess the basic and instrumental activities of daily living. The ranges of interpretation are ≤3 (normal), 4–6 (mild), 7–11 (moderate), and 12–33 (severe). Finally, the psychological subscale is composed of 10 items exploring symptoms of depression, psychosis, and anxiety. Scores are interpreted as follows: ≤5 (normal), 6–7 (mild), 8–11 (moderate), and 12–30 (severe). The original instrument was developed by Monahan et al. in 2012 to monitor dementia symptoms and subsequently validated to assess PICS [5,25].

### 2.5. Data Collection Procedure

Eligible participants were recruited in person during hospitalization. Discharged patients were identified via the institutional ICU database and contacted by phone to coordinate home visits. All visits adhered to a previously validated protocol, which included informed consent confirmation, stepwise explanation of study goals, uniform administration of instruments by trained research personnel, and immediate review of forms to minimize missing data.

Field teams underwent calibration sessions to ensure inter-rater reliability, and supervisors reviewed 10% of home assessments for quality control.

### 2.6. Statistical Analysis

Data were analyzed using IBM SPSS^®^ v28.0. Quantitative variables were described using mean ± standard deviation (SD) for normally distributed data or median and interquartile range (IQR) when non-normality was confirmed using the Kolmogorov–Smirnov test (*p* < 0.05 threshold). The choice to report means or medians was justified based on the skewness of the distribution. For instance, ICU length of stay and APACHE II scores followed a normal distribution (K-S *p* > 0.05), allowing the use of means and SD. Conversely, days on mechanical ventilation showed asymmetric distributions, justifying the use of medians and IQR.

Qualitative variables were summarized using absolute and relative frequencies. Bivariate analyses used Pearson correlation for continuous variables, chi-square test for categorical comparisons, and Kruskal–Wallis for variables with more than two groups.

Variables with *p* < 0.20 in bivariate analyses were entered into a multiple linear regression model (enter method). Assumptions tested included multicollinearity (VIF < 5), normality of residuals, and homoscedasticity. Covariates with *p* < 0.05 were retained as significant predictors.

### 2.7. Ethical Considerations

This research was endorsed by the Research Committee of the Faculty of Health Sciences of the University of Córdoba (Act number 06 of 14 August 2024) and was classified as minimal risk for the participants according to the provisions of Resolution 8430 of 1993 of the Colombian Ministry of Health [26]. To guarantee confidentiality and anonymity, each participant received an alphanumeric code irreversibly dissociated from any identifiable personal data. Likewise, informed consent was obtained from all participants prior to their inclusion.

## 3. Results

### 3.1. Sociodemographic and Clinical Characteristics

The sample analyzed was predominantly male (67.5%), with an average age of 56.2 years. A total of 35.3% had higher education, and most had a partner, were active workers (51.6%), resided in urban areas (74.3%), and professed the Catholic religion (67.5%).

Regarding the most frequent causes of admission to the ICU, 70% of the patients were hospitalized for conditions of cardiovascular or neurological origin. Regarding personal history, 66% had a previous diagnosis of arterial hypertension and type 2 diabetes mellitus. The mean ICU stay was 10.7 days, with a moderate mortality risk according to the APACHE II scale score.

Mechanical ventilation was used on 38.6% of the patients, with an average duration of 8.3 days, and 7.5% of the sample underwent tracheostomy. In relation to sedation, fentanyl was frequently used (38.6%) (Table 1).

### 3.2. Relationship Between Variables and HABC-M Scale

Regarding sociodemographic characteristics, statistically significant differences were identified in several variables. The religion variable showed an association with the severity of PICS (*p* = 0.001), indicating a relationship between religious affiliation and the degree of affectation. Similarly, significant differences were observed between groups according to educational level (*p* = 0.001), marital status (*p* = 0.001), and employment status, where a lack of employment and the role of full-time homemaker were related to lower scores on the HABC-M scale.

Regarding clinical variables, the analyses revealed statistically significant associations between the syndrome and the presence of personal history, the use of ventilatory support, the performance of tracheostomy, and the administration of cardiovascular or antibiotic treatments, all with values of *p* = 0.001 (Table 2).

To further investigate the relationship between clinical variables and the HABC-M score, boxplots were used. These graphs show a clinically consistent gradient in the severity of the post-intensive care syndrome, stratified into categories such as normal, mild, moderate, or severe according to the global score obtained.

In relation to age, a progressive shift in the median toward higher values was observed as the severity of the syndrome increased. In the group classified as normal, the median was around 40 years, while in the moderate and severe groups, it exceeded 65 years. In addition, the IQR was markedly widened, and both higher and lower outliers were identified, suggesting greater heterogeneity in older patients with more severe PICS.

The APACHE II scale—an indicator of acute severity at admission—shows an ascending pattern in the median score as the severity of PICS increases. Patients with no involvement present a median score close to 5 points, while in the severe category, it rises to values between 25 and 27 points. Likewise, the range of values widens progressively with severity, suggesting greater pathophysiological variability in the most compromised patients. The presence of only a few outliers at the extremes indicates that, in the most severe cases, even those at low risk according to APACHE II can develop significant involvement, while at the other extreme, some patients with high scores do not necessarily have severe PICS.

The early burden of care, as measured by the TISS-24 scale, showed a similar ascending pattern, albeit with a narrower absolute range. The groups classified as normal and mild shared a median of 2 points, while the moderate and severe levels reached medians of 3 to 4 points, with upper extremes of up to 4. These results suggest that, even within a limited range of intensity of care, patients who developed PICS, at moderate or severe levels, received more interventions during the first 24 h of ICU hospitalization on average.

**Figure 1:**
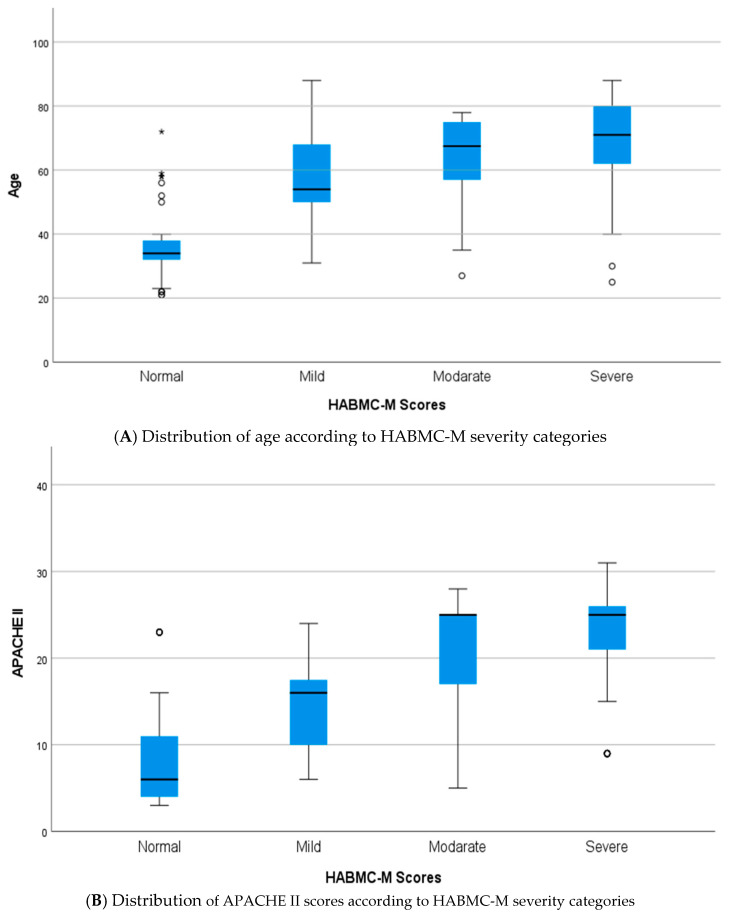

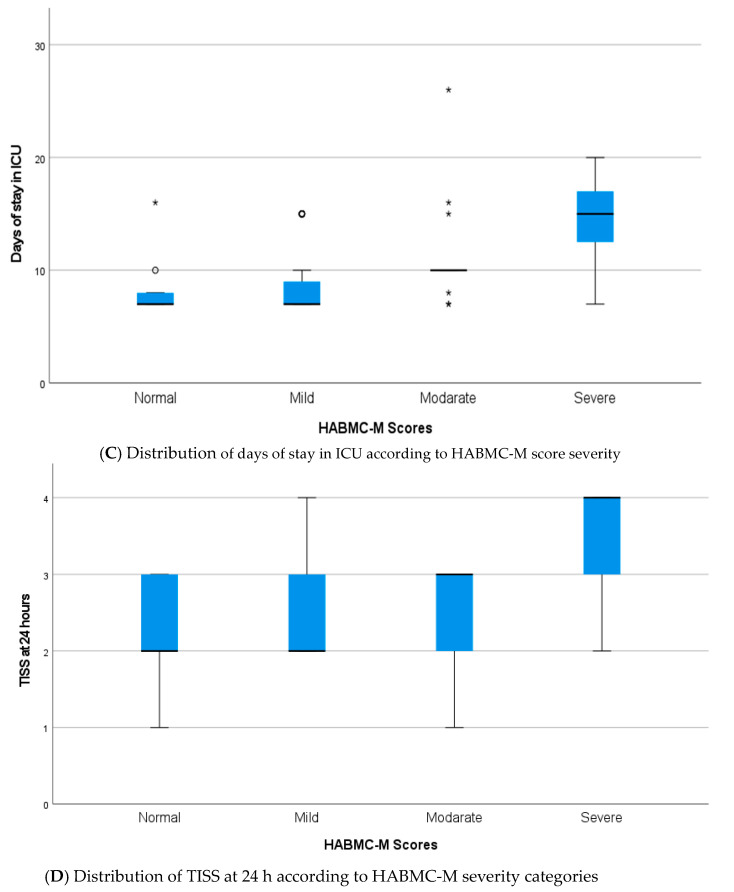
Boxplots showing the distribution of (**A**) age, (**B**) APACHE II scores, (**C**) days of stay in ICU, and (**D**) TISS scores at 24 h according to the severity categories of the HABMC-M scale (normal, mild, moderate, severe).

Finally, the greatest median increase was observed in the number of days of ICU stay. This rose from approximately 7 days in the unaffected group to 15 days in the group classified as severe. In addition, the boxplots showed an expansion in outliers of up to 20 to 30 days, highlighting that prolonged stay in intensive care, particularly following surgical interventions, is a distinctive feature in patients with greater overall functional impairment (Figure 1).

### 3.3. Correlation Analysis

A statistically significant positive correlation (*p* < 0.001) was observed between the overall HABC-M scale score and all quantitative clinical variables analyzed: age, TISS score at 24 h, APACHE II, length of ICU stay, and days of mechanical ventilation. The strongest association was identified between the overall HABC-M score and days of ICU stay (r = 0.79), followed by the TISS (r = 0.76) and APACHE II (r = 0.76) scores. Age presented a moderate correlation with the total scale score (r = 0.56). Likewise, days of mechanical ventilation showed a strong correlation with the global score (r = 0.69), which supports the existing evidence on the impact of prolonged ventilation on long-term neuropsychological and muscular dysfunction (Table 3).

The correlation matrix revealed positive associations between the HABC-M total score and all clinical variables analyzed. The strongest correlations were observed with the number of days in the ICU (r = 0.69), days on mechanical ventilation (r = 0.69), and the TISS score at 24 h (r = 0.61). As for the APACHE II score, a moderate correlation was evidenced with the HABC-M total score (r = 0.34), and a weaker correlation was observed with TISS (r = 0.20). The variable age showed a low correlation with the total score (r = 0.19), indicating a weak relationship between age and the severity of post-intensive care syndrome.

**Figure 2:**
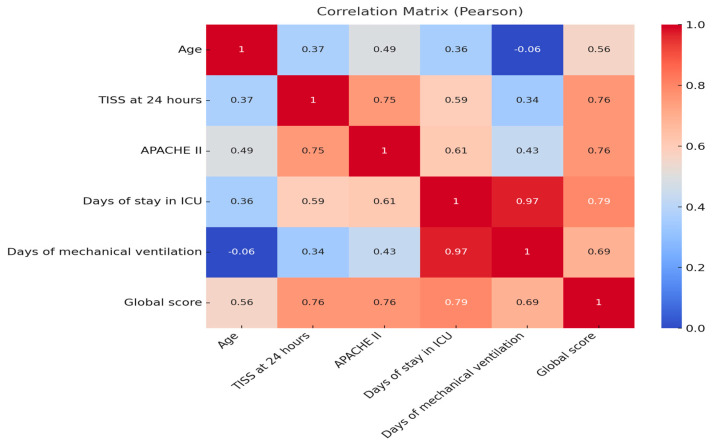
Correlation matrix (Pearson).

The clinical variables also showed a strong correlation between the number of days in the ICU and days on mechanical ventilation, with a very high correlation coefficient (r = 0.97), indicating a high degree of association between the two. In addition, moderate correlations were identified for APACHE II and time on ventilation (r = 0.43), as well as with length of ICU stay (r = 0.38). Similarly, the TISS score at 24 h showed moderate correlations with both length of ICU stay (r = 0.34) and days on mechanical ventilation (r = 0.34). Taken together, these findings support the observed trend that greater patient clinical complexity, as determined by TISS and APACHE II, together with the longer duration of mechanical ventilation and prolonged ICU stays, are associated with higher scores on the HABC-M scale, indicating greater post-intensive care syndrome severity (Figure 2).

Analysis using scatter plots with linear fit and 95% confidence intervals confirmed the positive correlation and facilitated visualization of the distribution pattern of cases, as well as the strength of each trend. A progressive increase with age was observed for the total HABC-M score. The slope of the fitted line was moderate, and the confidence interval remained narrow over most of the age range, indicating a relatively stable relationship. However, the scatter plot showed remarkable heterogeneity in the 45–70 age group, where both moderate and high total scores coexisted. This finding suggests that in this age group, other concomitant clinical variables that modulate the severity of PICS may influence its severity.

Given that only three discrete values (2, 3, and 4) are available, the figure shows a stepwise progression, with each increase in early care burden associated with a significant increase in the overall score. The slope, the steepest among the variables analyzed, is consistent with the high correlation observed (r = 0.76). The confidence interval widens at the extremes, reflecting the lower frequency of observations at TISS = 2 and 4. As is characteristic of ICU populations, the distribution shows a concentration of cases in the intermediate values (15–25 points). The positive slope indicates that a higher initial severity index is associated with more pronounced deterioration after discharge. The widening of the confidence interval below 10 and above 30 indicates lower precision at extremes due to the paucity of data.

**Figure 3:**
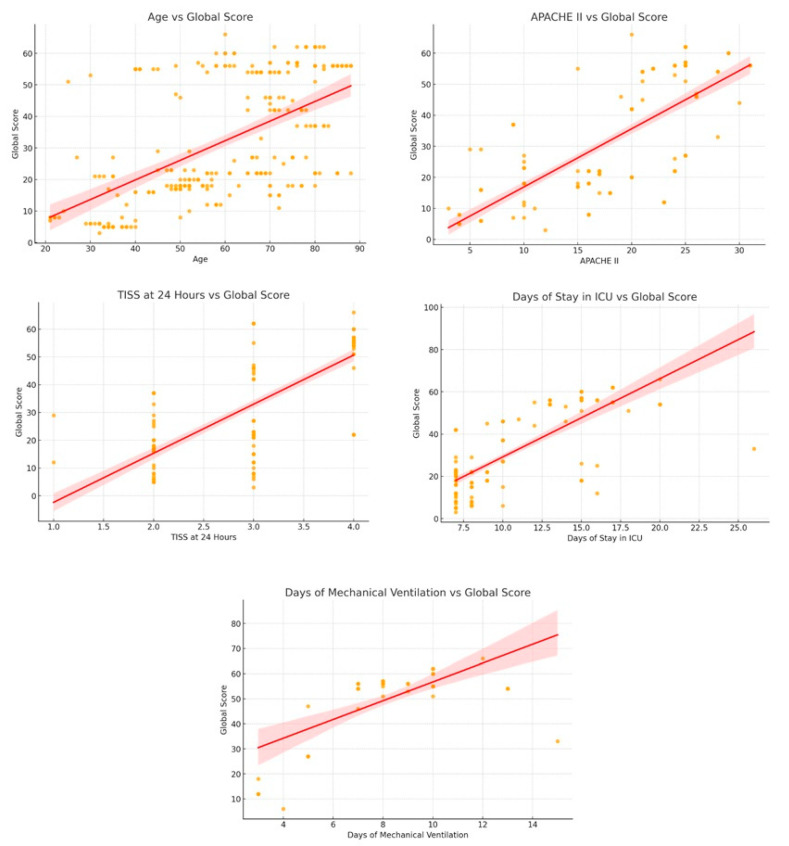
Correlation between clinical variables and global score in ICU patients. Correlation of the HABC-M score with the variables age, APACHE II, TISS at 24 hours, days of stay in the ICU and days of mechanical ventilation).

However, ICU days show a more pronounced linear relationship, with a slope indicating that for every five additional days of hospitalization, the overall HABC-M score increases by an average of 12 to 15 points. The confidence interval widens for stays longer than 20 days, reflecting greater variance due to clinical complications and comorbidities. For mechanical ventilation days, the pattern is similar, although there is greater dispersion and a wider confidence interval, suggesting greater inter-individual variability in response to ventilatory support. Nevertheless, the upward trend is clear: mechanical ventilation stays ≥10 days are consistently associated with total scores ≥ 60 (Figure 3).

### 3.4. Logistic Regression Model to Predict High HABC-M Scale Scores

When fitting a logistic regression model to predict a high score on the HABC-M scale, indicating greater symptomatic severity, we observed that cardiovascular treatment (β = 4.385; OR = 80.21; 95% CI: 1.46–7.31; *p* < 0.001) was significantly associated with high scores, as was the administration of antibiotics (β = 4.529; OR = 92.62; 95% CI: 2.21–6.85; *p* < 0.001). Fentanyl use showed a high coefficient (β = 12.840; OR = 376.962.13; 95% CI: 0.21–25.47; *p* = 0.05) with a particularly wide confidence interval, necessitating caution in its interpretation. By contrast, midazolam administration (beta = −4.960; OR = 0.01; *p* = 0.02) showed a protective effect against symptom severity.

Regarding sociodemographic factors, belonging to the evangelical religion (β = −11.874; OR ≈ 0.00; *p* < 0.001) and having completed primary school (β = −3.223; OR = 0.04; 95% CI: 0.00–0.57; *p* = 0.02) were associated with a higher probability of scoring high on the scale. Similarly, living in an urban area (β = −3.199; OR = 0.04; *p* = 0.02) was associated with a lower risk. By contrast, not being employed (β = 4.802; OR = 121.71; *p* = 0.01) significantly increased the odds of having higher symptom severity, whereas being single (β = −5.314; OR ≈ 0.00; *p* < 0.001) showed a protective effect.

For each additional year of age (beta = 0.291; OR = 1.34; 95% CI: 1.25–1.44; *p* < 0.001), the odds of scoring high on the scale increased by 34%. Similarly, a higher score on the TISS scale at 24 h (β = 8.317; OR = 4093.25; *p* < 0.001) and on the APACHE II scale (β = 0.511; OR = 1.67; 95% CI: 1.35–2.06; *p* < 0.001) was associated with a higher likelihood of developing severe sequelae. Finally, each additional day of ICU stay (β = 2.321; OR = 10.19; 95% CI: 7.75–13.40; *p* < 0.001) was associated with a tenfold increase in the odds of scoring highly on the HABC-M scale (Table 4).

## 4. Discussion

This research aimed to identify the factors related to post-intensive care syndrome in a sample of hospitalized patients. In this sense, statistical analyses established a statistically significant correlation between some sociodemographic characteristics—as well as with most of the selected clinical variables—and the presence of PICS, providing relevant information on the behavior of this phenomenon.

The logistic regression model fit showed that living in an urban area and having attended only primary school were associated with a lower likelihood of scoring high on the HABC-M scale, which assesses the presence and severity of PICS, a finding consistent with a report by Haddad et al. [27].

Regarding the statistically significant association of variables such as urban residence and evangelical religion with a lower risk of presenting with the syndrome, no previous studies were identified that allowed us to directly contrast these findings. However, some research agrees that meeting the spiritual needs of critically ill patients as part of prevention and treatment strategies for PICS can reduce its incidence and alleviate the severity of symptoms [28,29]. This is a possible explanation for this finding. However, unemployment was associated with more severe cases of the syndrome, a finding consistent with previous research documenting an association between this variable and significant cognitive changes at 3 and 12 months after discharge from the ICU [27,30].

Single marital status showed a protective effect against this condition. Although no studies were found that directly contradicted this result, the findings of authors such as Shirasaki et al. [31], in an integrative review, contradict the results of this study, stating that marital status is not directly related to post-intensive care syndrome. However, this characteristic may influence the availability of social and emotional support during and after hospitalization, which has been associated with a lower likelihood of developing PICS [32].

Regarding the medical therapy applied, we established a statistically significant relationship between invasive mechanical ventilation support and the presence of PICS. In this sense, mechanical ventilation has been reported as a factor associated with this syndrome in surviving critically ill patients according to previous studies that specifically analyzed the behavior of this variable [33,34].

Similarly, the performance of tracheostomy also showed a statistically significant association with higher scores on the scale assessing this syndrome, which is consistent with that reported by Iribarren et al. [35]. However, this finding differs from that of another study, which found no statistically significant association between this procedure and the development of PICS [36]. This discrepancy highlights the need for long-term follow-ups of this population to clarify the true impact of tracheostomy on the incidence and severity of post-intensive care syndrome.

Another relevant finding was the greater probability of developing post-intensive care syndrome in patients who received cardiovascular support drugs or antibiotics. In this regard, no studies were identified that directly contrasted with this result. However, in several systematic reviews, the administration of these pharmacological groups was not included as a risk factor associated with the syndrome [37,38]. That said, the use of midazolam seemed to exert a possible protective effect against PICS in the sample analyzed, in contrast to what was reported by Martínez et al. [39], who observed greater physical sequelae in patients who received accumulated doses of this drug.

When analyzing variables such as age and length of hospital stay, the data suggest that both older age and each additional day spent in the ICU increase the likelihood of not only developing PICS but also its more severe forms. The association of these two variables with PICS has been extensively documented in previous research investigating predisposing factors for the occurrence of sequelae after critical illness [40,41], which is consistent with the findings of the present study and contributes to its validation.

The correlation matrix of variables suggested that a personal history of arterial hypertension, diabetes, or chronic obstructive pulmonary disease can be configured as a set of risk factors for the development of post-intensive care syndrome. This finding is consistent with that of Kang et al. [12], who identified these antecedents as predictors of physical, mental, and cognitive sequelae in patients after discharge from the ICU.

Patients with neurological diagnoses at the time of ICU admission were found to have more severe forms of PICS. In this regard, previous studies have only implicated delirium as a predisposing factor for the development of PICS [4,42], and no research was found that directly contradicted this finding. However, the literature points out that studies focusing on PICS often exclude patients with acute brain injury or previous chronic neurodegenerative diseases, making it difficult to differentiate between sequelae due to pre-existing conditions and those due to intensive care [8]. This limitation suggests the need to explore new phenomena of interest to the discipline of nursing.

Our results show that post-intensive care syndrome continues to be a public health problem of special interest for nursing, considering the fundamental role that nurses play in the care processes for the prevention, treatment, rehabilitation, and reincorporation of the person who suffers from it.

The configuration of PICS as a multifactorial phenomenon, as demonstrated in the results herein, implies that nursing professionals should have updated information that facilitates the design of interventions aimed at comprehensively managing this condition.

### Limitations and Implications for Nursing

Post-intensive care syndrome has become a relevant challenge for nursing practice. Its high incidence among patients who survive an ICU stay demonstrates the need to understand its behavior and the variables that determine it. The results of this study have important implications for the care of critically ill patients, as they provide an overview of the main predictors of PICS, which can be considered in formulating preventive interventions and in orienting future research aimed at deepening the understanding of this phenomenon. It is also essential to raise awareness among the healthcare team of the importance of recognizing and preventing this syndrome.

Our literature review also identified significant theoretical gaps related to post-intensive care family syndrome (PICS-F). Most research has focused on the patient, ignoring the emotional, physical, and economic impact that family members experience when a member is critically ill. This situation has direct implications in clinical practice, considering the fundamental role of the family in the recovery process of the critically ill patient. Therefore, there is a need to conduct research aimed at understanding the behavior of PICS-F and to propose care strategies focused on preventing this syndrome among family members.

Despite the relevance of these results, it is important to recognize certain limitations in the study. First, because this was not a prospective study, it was impossible to determine with certainty the influence of long-term sociodemographic and clinical characteristics on the patients included in the sample. Another possible limitation is the type of sampling used. Because non-probability sampling was used, it is possible that some patients with valuable information regarding risk factors for post-acute care syndrome were excluded.

Finally, the results should be interpreted with caution because the study was based on a purposive sample collected in a specific geographic location, which may limit the generalizability of the results to other populations with different characteristics.

## 5. Conclusions

Post-intensive care syndrome (PICS) continues to be a significant health problem among patients discharged from intensive care units (ICUs). In the sample analyzed, a statistically significant association was found between PICS and several sociodemographic variables, such as age, urban residence, religious affiliation, and unemployment. However, the clinical variables most strongly associated with this syndrome included mechanical ventilation, longer ICU stays, the presence of comorbidities, tracheostomy, the administration of sedatives and cardiovascular medications, and the severity of illness. Further research is needed to evaluate other predictors of PICS, as well as the behavior of this phenomenon in families: post-intensive care syndrome families.

Finally, these results have implications for nursing practice, specifically in the design of interventions that can identify and prevent post-intensive care syndrome.

## Figures and Tables

**Table 1:** Sociodemographic and clinical characteristics.

Variable	Category	*n*	%	Mean/SD *
**Age (years)**				59.2 ± 17.3
Sex				
	Male	187	67.5	
	Female	90	32.5	
Marital status				
	Single	135	48.7	
	Married	127	45.8	
	Other/widowed †	15	5.4	
Educational level				
	Primary	71	25.3	
	Baccalaureate	82	29.6	
	Higher education	98	35.3	
	Technical studies	26	9.4	
Employment status				
	Worker	143	51.6	
	Does not work	73	26.3	
	Housewife	56	20.2	
	Student	5	1.8	
Religion				
	Catholic	187	67.5	
	Evangelical	80	28.8	
	Not professed	10	3.6	
Provenance				
	Urban	206	74.3	
	Rural	71	25.6	
Clinical conditions of admission to the ICU due to altered systems				
	Cardiovascular	81	29.2	
	Cardiovascular + neurological	59	21.2	
	Neurological	53	19.2	
	Respiratory	42	15.1	
	Other/mixed	42	15.1	
Personal Antecedents				
	AHT	82	29.6	
	AHT + DM2	64	23.1	
	COPD	44	15.8	
	DM2	34	12.2	
	Other/none	53	19.1	
APACHE II				17.3 ± 7.8
Days of stay in ICU				10.7 ± 4
Sedatives				
	Fentanyl	107	38.6	
	Midazolam	64	23.1	
	Dexmedetomide	44	15.8	
Mechanical ventilation				
	Yes	107	38.6	
	No	170	61.3	
Mechanical ventilation days				8.3 ± 2.5
Tracheostomy				
	Yes	21	7.6	
	No	256	92.4	

* SD: Standard deviation. † Includes separated, widowed, or undeclared common-law spouse.

**Table 2:** Relationship between variables and HABC-M scale.

		Total Score HABC-M Scale				
Variable	Category	Normal	Slight	Moderate	Severe	*p*-Value
Religion	Catholic	33	63	4	87	0.001
	Evangelical	8	31	12	29	
	Not professed	5	5	0	0	
Educational level	Primary	1	30	2	38	0.001
	Baccalaureate	14	30	11	27	
	Technical studies	1	21	2	2	
	Higher education	30	18	1	49	
Marital status	Single	31	53	3	48	0.001
	Married	11	35	13	68	
	Free union	4	11	0	0	
Employment status	Housewife	1	17	0	38	0.001
	Worker	40	75	4	24	
	Does not work	0	7	12	54	
	Student	5	0	0	0	
Clinical conditions of admission to the ICU due to altered systems	Neurological	2	20	0	31	0.001
	Cardiovascular	5	42	4	30	
	Respiratory	5	9	1	27	
	Gastrointestinal	11	12	0	1	
	Cardiovascular + neurological	16	6	11	26	
	Endocrine	7	0	0	0	
	Kidney	0	10	0	1	
Personal antecedents	AHT + DM2	3	28	12	21	0.001
	DM2	6	4	1	23	
	AHT	13	32	2	35	
	DM1	7	0	0	0	
	CKD	0	0	0	7	
	AHT + DM2 + CKD	1	19	0	1	
	COPD	5	10	0	29	
	Other	11	6	1	0	
Mechanical ventilation	Yes	6	1	11	89	0.001
	No	40	98	5	27	
Tracheostomy	Yes	0	0	1	20	0.001
	No	46	99	15	96	
Cardiovascular treatment	Yes	4	87	14	115	0.001
	No	42	12	2	1	
Nitroglycerin	No	41	55	11	87	0.001
	Yes	2	44	3	29	
Nitroprusside	No	46	84	16	106	0.001
	Yes	0	15	0	10	
Norepinephrine	No	43	87	6	40	0.001
	Yes	0	12	10	75	
Dopamine	No	46	98	16	49	0.001
	Yes	0	1	0	66	
Labetalol	No	46	97	15	116	0.087
	Yes	0	2	1	0	
Sedatives	Yes	7	18	12	99	
	No	39	81	4	17	
Dexmedetomide	No	46	88	4	96	0.001
	Yes	0	11	12	20	
Fentanyl	No	40	97	5	27	0.001
	Yes	6	2	11	89	
Midazolam	No	45	93	16	58	0.001
	Yes	1	6	0	58	
Diazepam	No	45	93	16	115	0.001
	Yes	1	5	0	0	
Antibiotic treatment	Yes	10	39	2	76	0.001
	No	36	60	14	40	
Piperacillin_tazobactam	No	46	71	15	68	0.001
	Yes	0	28	1	48	
Cefepime	No	45	88	15	115	0.001
	Yes	1	11	1	1	
Ampcillin_sulbactam	No	46	89	16	89	0.001
	Yes	0	10	0	27	
Clarithromycin	No	46	99	16	96	0.001
	Yes	0	0	0	20	
Others	No	38	91	16	78	0.001
	Yes	8	8	0	38	

**Table 3:** Correlation analysis.

	Pearson Correlation	CI 95%		
		Lower	Upper	*p*-Value
Global score—Age	0.56	0.47	0.64	0.00
Global score—TISS at 24 h	0.76	0.70	0.80	0.00
Global score—APACHE II	0.76	0.71	0.81	0.00
Global score—Days of stay in ICU	0.79	0.74	0.83	0.00
Global score—Mechanical ventilation days	0.69	0.57	0.78	0.00

**Table 4:** Regression model.

			CI 95%		
Variable	Coefficient	OR	Lower	Upper	*p*-Value
Received cardiovascular treatment (Yes)	4.385	80.21	1.46	7.31	0.00
Received antibiotic treatment (Yes)	4.529	92.62	2.21	6.85	0.00
Fentanyl (Yes)	12.840	376,962.13	0.21	25.47	0.05
Midazolam (Yes)	−4.960	0.01	−9.02	−0.90	0.02
Religion (Evangelical)	−11.874	0.00	0.00	0.00	0.00
Educational level [T. Primary]	−3.223	0.04	0.00	0.57	0.02
Provenance [T. Urban]	−3.199	0.04	0.00	0.63	0.02
Employment status [T. Does not work]	4.802	121.71	3.34	4435.53	0.01
Marital Status [T. Single]	−5.314	0.00	0.00	0.04	0.00
Age	0.291	1.34	1.25	1.44	0.00
TISS at 24 h	8.317	4093.25	565.12	29,648.21	0.00
APACHE II	0.511	1.67	1.35	2.06	0.00
Days of stay in ICU	2.321	10.19	7.75	13.40	0.00

## Data Availability

The data presented in this study are available from the corresponding author upon request.

## Public Involvement Statement

No public participation was undertaken in this study.

## Guidelines and Standards Statement

This manuscript was written in accordance with STROBE guidelines.

## Abbreviations

The following abbreviations are used in this manuscript:

ICU	Intensive Care Unit
SCCM	Society Critical Care Medicine
PICS	Post Intensive Care Syndrome
APACHE II	Acute Physiology and Chronic Health Evaluation II
HABC-M	Healthy Aging Brain Care Monitor
SD	Standard Deviation
IQR	Interquartile Range
COPD	Chronic Obstructive Pulmonary Disease
CI	Confidence Interval
OR	Odds Ratio
PICS-F	Post Intensive Care Family Syndrome
DM2	Type 2 Diabetes Mellitus
DM1	Type 1 Diabetes Mellitus
AHT	Arterial Hypertension
CKD	Chronic Kidney Disease
